# Life as a chief of medicine—Taking future hospital guidance to the front line

**DOI:** 10.1016/j.fhj.2024.100149

**Published:** 2024-07-12

**Authors:** Dr Ben Mearns

**Affiliations:** Surrey & Sussex Healthcare NHS Trust, Canada Avenue, Redhill, Surrey RH1 5RH, United Kingdom

**Keywords:** Leadership, Future hospital, Chief of Medicine, Values, Quality improvement

## Abstract

The Future Hospital Commission developed a comprehensive and ambitious proposal of how hospitals should be run, with clinical leadership at its heart. A medical division led by the chief of medicine was integral to the vision and these senior physicians would be central to the running of hospitals alongside managers. The real-world experiences over 8 years of one chief of medicine are explored and compared to the guidance set out by the Future Hospital Commission, with an emphasis on interdisciplinary triumvirate working, values, behaviours, quality improvement and the impact of crises like the COVID pandemic. The chief of medicine role can play an important part in delivering the outcomes promoted by the Future Hospital Commission, but the role is likely to vary throughout the NHS dependent on local factors. Future guidance could focus on equipping the leaders of the future to design and improve services, and to develop the multiprofessional teams that meet the needs of their specific challenges that they face.

## The future hospital

The Future Hospital Commission developed a future hospital vision, made up of 50 recommendations forming a comprehensive and ambitious proposal of how hospitals should be run, with clinical leadership at its heart.^1^

The principles underpinning the future hospital vision were based on solid ground, with clear expectations on care for patients and medical professionalism.

From this foundation, the commission presented a vision for a new model of clinical care, focused on the needs of patients with continuity of care by effective teams at its heart. The principles recognised the balance between excellent general internal medicine and access to specialty advice whenever it was required.

A new organisational approach was suggested to bring together medical services and staff into a single medical division under the new role of chief of medicine, assisted by an acute care coordinator and a chief resident – to become the successful Chief Registrar Programme.^2^

The vision for the chief of medicine was a post covering all clinical services, including surgical, facing both into and outside of the hospital. The recommendation was extremely bold and novel for the NHS.

In reality, the chief of medicine in this vision, would be an important deputy to the medical director in terms of quality, safety, clinical effectiveness and medical professionalism as the role took on leading medical care throughout the hospital. It would also be a key role in operational management and responsible to the chief operating officer in terms of performance and responsiveness. It was a post that would occupy a space between executive directors and the clinical directors in most hospitals, and essential in strategic planning: quite a big job.

## My experience as a chief of medicine

In 2015 I became chief of medicine for Surrey & Sussex Healthcare (SaSH) NHS Trust, a post with significant differences, and similarities, to the post envisaged by the Future Hospital Commission.

Our trust had recently completed work with GE Healthcare to create a ‘capable, strong and innovative team of leadership-focused clinicians who would drive strategy and create strong and resilient relationships with general and nurse management colleagues’.^3^ This work created a clinically led and management-enabled approach, recognising the differences between clinical leadership and management and creating teams with the capability to run the hospital's services together.

The link between leadership and good clinical and operational outcomes has been well-described.^4^

**Fig. 1:**
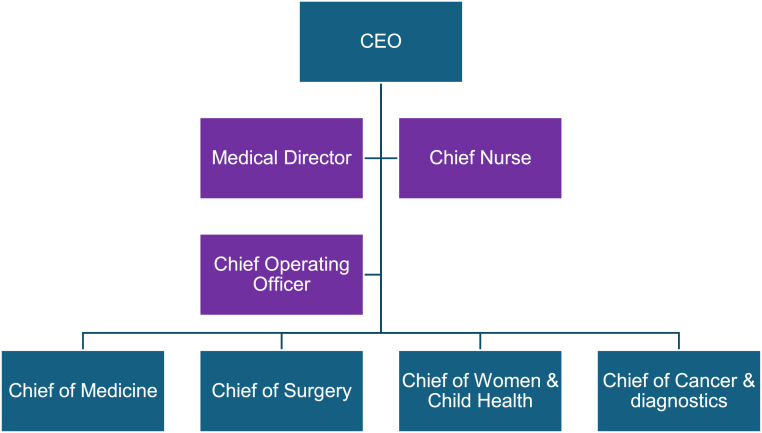
Organisational chart of senior medical leadership at surrey & sussex healthcare NHS trust from 2015. The board level medical, nursing and management triumvirate is shown in purple.

**Fig. 2:**
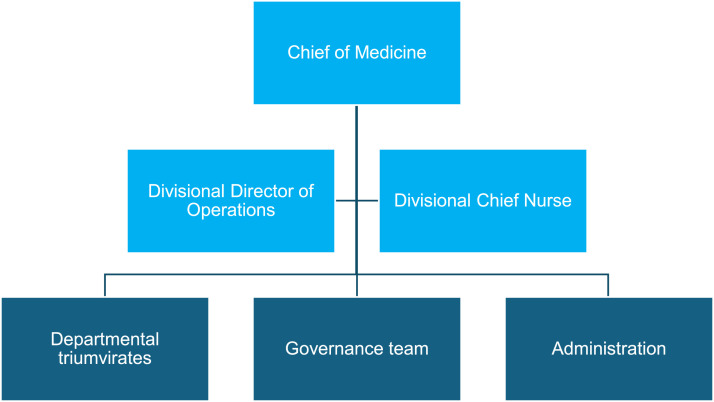
Organisational chart of medicine division senior leadership and management support at surrey & sussex healthcare NHS trust from 2015. The divisional level medical, nursing and management triumvirate is shown in light blue.

Clinical chief posts were created to lead four divisions as shown in Fig. 1. The chiefs were voting members on the Executive Committee of the trust, line managed by the CEO, professionally responsible to the medical director and led their services alongside a senior manager and divisional chief nurse as a triumvirate Fig. 2.

The triumvirate for departments brought together clinical leads, matrons and service managers. Lines of professional accountability flowed along the disciplines of medicine, nursing and management, but in terms of overall responsibility, this followed the medical line.

The triumvirates operated as teams, and from their formation everything that could be attributed to an individual, was a team effort. The key to effectiveness came from the team working together, with good relationships and complimentary skills and experience.

## Values and behaviours

Our trust's leadership knew that culture was key to improvement, and had recently launched values that everyone could believe in. Everyone at the trust was able to contribute to their creation and they became important to everyone as a result. The values are safety & quality, compassion, dignity & respect and #OneTeam, all designed to ‘put people first’.^5^ Each of the values was rooted in ten ‘behavioural anchors’, a description of the behaviours expected by our team to ensure those values were in place, with examples given of good and bad behaviours.

Performance reviews were created for all staff that reflected not only performance, but crucially our newly shared values and behaviours, with a view to promote role models, and to address behaviours that were not acceptable. Leaders were appointed based on values and behaviours as much as on their performance.

In our organisation we felt strongly that if you focused on the values, then everything else would flow from that.

## Quality improvement

I was appointed into post shortly before the start of a 5-year partnership with the Virginia Mason Institute (VMI)^6^ and my time as chief was defined by this.

On our trip to Seattle, we had chiefs alongside key members of the executive team, learning together and getting to know each other. On our return, this Trust Guiding Team met regularly, and meetings were held once a month with a requirement for all to attend. They were structured, and the pace controlled to allow us to develop relationships, to think slowly^7^ and become more effective together.

We created our SaSH+ lean management system^8^ with the help of VMI. A core part of the system is for senior leaders to visit all of their teams in the place where the work is done (gemba in Japanese). Our divisional triumvirate would visit each clinical area on a rotating schedule and engage with all staff on the ward. We would ‘go see’ and ‘find out’ and assist our teams who were doing the work to improve the work.^9^ It was the most effective use of our time, brought the triumvirate together and firmed up our relationships and with multiple members of our clinical and managerial teams.

Staff of all grades from all services had been trained alongside each other on the Lean for Leaders course. The students on each course would learn the SaSH+ management system, form friendships and work with colleagues that they would never have done so before. Hierarchy was removed, and teams were reaching out across the usual boundaries to get things done. The Warwick business school evaluation of the VMI work showed a strikingly different social network to other trusts with a high degree of connectivity and interaction. ‘Everyone is talking to each other’.^10^ It really did feel like that and the benefits were very evident to see.

Over time, and with deliberate effort, the values, behaviours, and the new management system became truly embedded and in 2018 the CQC remarked on the ‘compassionate, inclusive and effective leadership at all levels’ and the ‘skilled, stable and highly visible senior management team that possessed a deep understanding of issues, challenges and priorities affecting their service.’^11^

## The COVID pandemic

There are many lessons to learn from the COVID pandemic and the enquiry is ongoing.^12^

My clinical job moved to COVID full time alongside colleagues, and my leadership role continued alongside. There were so many decisions to be taken so quickly, that I certainly became more reactive and acted as a problem-solver rather than the more effective problem-framer I had become. As our colleagues throughout the NHS did, we met the challenge together as a team and delivered the very best services for patients that we could.

The three principles that I led with during the pandemic were to keep clinical teams together, keep them doing what they're trained to do, and for us all to be forgiving of ourselves and each other when we didn't get it right. Certainly, the first two principles were part of the vision for a new model of care in future hospital,^1^ but the third was likely the most important and the one I needed the most as chief of medicine.

Psychological trauma^13^ and mental exhaustion were common in our team, including myself. I didn't recover properly until I had stood down as chief and allowed myself time to rest.

## Comparison of my experience versus future hospital

There are many similarities between my experience and the principles and vision set out by the Future Hospital Commission, but there are differences in my experience as shown in Table 1.

**Table 1:** Differences in the experience of a Chief of Medicine to the principles and vision set out by the future hospital commission.

	Future hospital	My experience
Foundation	Principles	Values
Main focus	Clinical services	People
Strategy	Transformation	Quality improvement
Scope	Comprehensive	Focussed
Chief of medicine	Trust-wide medical leader	Leader of the division

The unique circumstances that the hospital and the people running it were experiencing at the time of the Future Hospital Commission report must drive the response. We had to focus on values and behaviours, and to build a culture of improvement within the executive team, clinical leaders and managers to then develop a highly effective team of leaders throughout our organisation. Our strategy was to learn and deploy quality improvement at pace and scale to empower our team to focus their efforts on the improvements that needed to be done to meet our newly shared values.

Many of the improvements made were those listed in the Future Hospital Commission, including a new and expanding general internal medicine department, enhanced 7-day services, integrating more with our community, and of course the creation of a Medicine Division and chief of medicine, albeit as one of four clinical divisions rather than covering all medical disciplines. Our operational structures may have remained different to those suggested, but appeared to be similarly effective.

## Discussion

My experience as a chief of medicine is unique, and this will be the case for others. The circumstances, challenges, people and culture will all be different at the time a physician steps up to the role and this will inevitably define the response required. It is unlikely that one model can ever apply to the individual needs of each organisation.

If I had to sum up what I think works, I would say that running hospitals is all about people and their relationships. With good relationships, comes honesty, psychological safety, permission to lead, and an understanding of how the team can solve problems together. Leaders should be equipped with the training and support required to foster a collaborative culture based on positive relationships.

Promoting people into clinical leadership based on values and behaviours will always be right, and thought should always be given to how the wider leadership team fits together into a highly functioning group with a comprehensive skill set, relevant experience and shared purpose.

The Future Hospital Commission's recommendations were an excellent example of how you could run a hospital, based on sound principles, but it was directive in my view, which could limit application. We must recognise the complexity and variability of medical services across hospitals^14^ and while one solution is attractive, the journey to achieve it may be too far to reach in the short term. To meet all challenges, it may be that developing future guidance for senior clinical leaders should prioritise values, behaviours and quality improvement.

It takes time to build the conditions for successful transformation, and its hard work. Guidance to clinicians and trusts on how to develop their teams and the environment in which they all work could enable transformational work later. Investing in the development of clinical leaders is an important part of this, and should be prioritised, building on the work of the Future Hospital Commission.

## Conclusion

The chief of medicine can play an important part in delivering the outcomes promoted by the Future Hospital Commission. The role is likely to vary throughout the NHS, and future guidance could focus on equipping the leaders of the future to design and improve services, and to develop the multiprofessional teams that meet the needs of the specific challenges that they face.

## CRediT authorship contribution statement

**Dr Ben Mearns:** Writing – original draft, Writing – review & editing.

## Declaration of competing interest

The authors declare that they have no known competing financial interests or personal relationships that could have appeared to influence the work reported in this paper.
